# Sea Anemone Toxins: A Structural Overview

**DOI:** 10.3390/md17060325

**Published:** 2019-06-01

**Authors:** Bruno Madio, Glenn F. King, Eivind A. B. Undheim

**Affiliations:** 1 Institute for Molecular Bioscience, The University of Queensland, St Lucia, QLD 4072, Australia; glenn.king@imb.uq.edu.au; 2 Centre for Advanced Imaging, The University of Queensland, St. Lucia, QLD 4072, Australia; e.undheim@uq.edu.au; 3 Centre for Ecology and Evolutionary Synthesis, Department of Biosciences, University of Oslo, 0316 Oslo, Norway

**Keywords:** sea anemone, venom, toxin, molecular scaffold, neurotoxin, cytotoxin, enzyme

## Abstract

Sea anemones produce venoms of exceptional molecular diversity, with at least 17 different molecular scaffolds reported to date. These venom components have traditionally been classified according to pharmacological activity and amino acid sequence. However, this classification system suffers from vulnerabilities due to functional convergence and functional promiscuity. Furthermore, for most known sea anemone toxins, the exact receptors they target are either unknown, or at best incomplete. In this review, we first provide an overview of the sea anemone venom system and then focus on the venom components. We have organised the venom components by distinguishing firstly between proteins and non-proteinaceous compounds, secondly between enzymes and other proteins without enzymatic activity, then according to the structural scaffold, and finally according to molecular target.

## 1. Introduction

**Figure 1:**
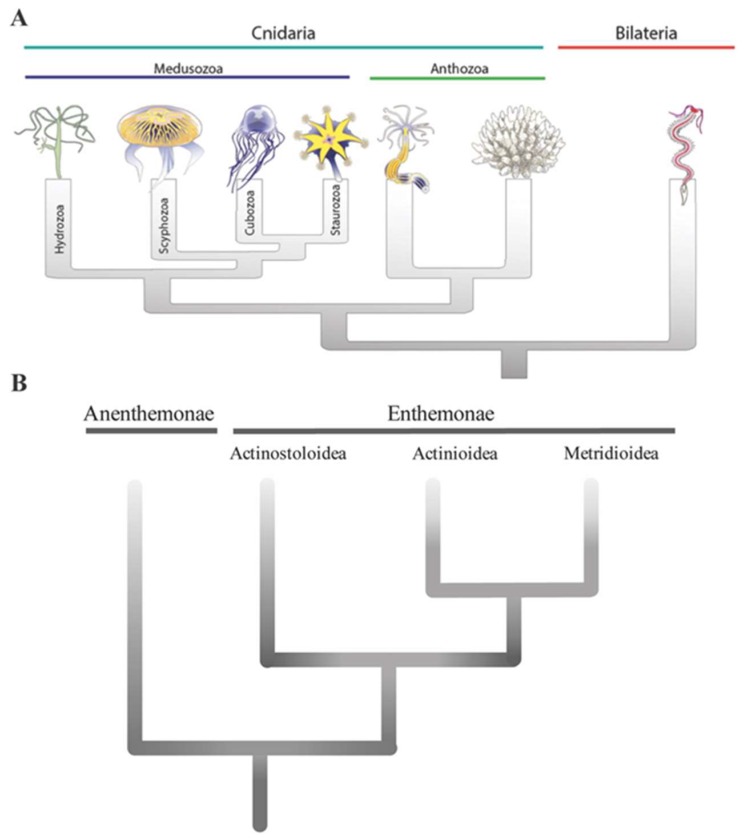
(**A**) Phylogenetic tree of Cnidarians. Representative Medusozoa depicted here are: *Hydra viridis* (Hydrozoa), *Aurelia aurita* (Scyphozoa), *Chironex fleckeri* (Cubozoa), and *Haliclystus sp* (Staurozoa). Representative Anthozoa are the sea anemone *Nematostella vectensis* (left) and the coral *Acropora millepora* (right). Figure modified with permission from Marine Genomics; published by Elsevier, 2015 [9]. (**B**) Phylogenetic relationships among major lineages of sea anemones (after Rodrigues et al., [4]).

Sea anemones, sometimes poetically referred to as the flowers of the sea, are exclusively marine animals that belong to the phylum Cnidaria (Figure 1A). Essentially laminar organisms, their two-dimensional epithelial construction has shaped their behavioural and physiological responses and has led to great ecological success despite their structural simplicity, as evidenced by their presence in all marine ecosystems. Sea anemones also play an important role in benthic–pelagic coupling as part of the benthic suspension feeding community [1], transferring energy to the benthos from the water column and releasing metabolites, gametes, and offspring back into the water column.

Sea anemones belong to the class Anthozoa, which differ from all other cnidarians in that they lack a free-swimming medusa stage. Within Anthozoa, sea anemones form the hexacorallian order Actiniaria, which contains only solitary, sessile, benthic polyps. There are around 1200 species of sea anemones organized in 46 families and they constitute the greatest diversity within Anthozoa. Polyps may be single sex or hermaphrodites, and they can reproduce either sexually of asexually. The sexual life cycle is straightforward, and includes four main stages: fertilized egg, planula, polyp, and adult. Sea anemones have great powers of regeneration [2], and can reproduce asexually in multiple ways: by budding, fragmentation, or by longitudinal or transverse binary fission [3].

Relationships within Actiniaria as determined by phylogenetic analyses of DNA or morphological characters do not accord with the divisions of the traditional classification, and thus the order was recently revised to resolve this conflict [4]. The primary division within the order is between the Anenthemonae and Enthemonae. Anenthemonae is the less speciose suborder, containing members of the families Actinernidae, Edwardsiidae, and Halcuriidae. The model organism *Nematostella vectensis* is the most familiar and well-studied member of this group. Enthemonae contains the overwhelming majority of species and anatomical diversity within Actiniaria and it is further subdivided into the superfamilies Actinioidea, Actinostoloidea, and Metridiodea (Figure 1B).

Although sea anemones are flexible in the ways in which they obtain nutrition [5], they are fundamentally predatory animals, using their tentacles to catch prey. Because they lack true muscle tissue, have no visual capacity, and lack a centralized or coordinated nervous system, sea anemones rely heavily on toxins for prey capture. The dietary composition of species varies markedly between different marine habitats, reflecting the different composition of the macrobenthic organismic assemblages in different areas [6]. Sea anemones capture prey that come within reach of their tentacles, enabling them to immobilize the prey with their venom. The mouth can stretch to help in prey capture and ingestion of larger animals such as crabs, molluscs and even fish [7]. Sea anemones are commonly considered a group of exclusively predatory animals, however they are also opportunistic, omnivorous suspension feeders. Some sea anemones feed to a large extent on organic detritus, which is caught with the aid of a mucus secretion. In addition, many sea anemones form a facultative symbiotic relationship with zooxanthellae, zoochlorellae, or both. These single-celled algal species reside in the anemone’s gastrodermal cells, especially in the tentacles and oral disc. The sea anemone benefits from the products of the algae’s photosynthesis and the algae in turn are assured protection and exposure to sunlight [8].

## 2. Venom Tissue

Cnidarians represent the only lineage of venomous animals that lack a centralized venom system. Instead of a venom gland, sea anemones produce venom in tissues throughout the body using two different type of cells, known as nematocytes and ectodermal gland cells [10,11]. Nematocytes, which are present in all cnidarians, produce highly complex venom-filled organelles known as nematocysts. Nematocysts are the primary venom delivery apparatus of cnidarians, and they are made of a capsule containing an inverted tubule capable of extremely fast and powerful discharge [12,13]. There are at least 25 different types of nematocysts in sea anemones, with multiple types harboured by a single specimen [14]. Moreover, distinct morphological regions of a sea anemone have specialised structures and they are defined by a specialised complement of nematocysts [15]. Examples of functional specialisation of the venom in different tissues includes tentacles used for prey capture, immobilisation and defence; acrorhagi used for competition and defence; column used for external defence; and actinopharnyx and mesenterial filaments, both used in prey immobilisation and digestion [16]. The ecological and evolutionary success of cnidarians since the Cambrian explosion may be due in large part to this complex organellar system and the toxins it contains.

In addition to nematocytes, sea anemones also produce toxins in a second type of cell known as an ectodermal gland cell, which may or may not produce distinct repertoires of toxins compared to nematocysts [10,17]. The reason why sea anemone toxins are located in two different types of cells remains unknown. However, secretion by gland cells may allow for delivery of larger amounts of the toxin, and present an opportunity to extend the reach of venom use by the anemone beyond direct contact. So far, gland cells have only been reported to be present in *Anthopleura elegantissima*, *Anemonia viridis*, and *Nematostella vectensis* [10]. However, given the wide phylogenetic distribution of these species, gland cells are likely to be present in most if not all sea anemones.

In general, most sea anemones are harmless to humans. Most sea anemones envenomations only cause skin rashes and edema in the area of contact with the tentacles. However, the venom of some species from the genera *Actinodendron*, *Telmatactis*, *Phyllodiscus* and *Triactis* can cause severe effects such as acute pain, necrosis, cardiotoxicity and neurotoxicity [18]. Envenomation sequelae may be linked to the size and types of nematocysts. For example, the extremely large basitrich nematocysts found in the balloon-like extensions of branching tentacles (acrospheres) of some sea anemones [19] may be capable of penetrating the epidermis, explaining the severe symptoms observed in humans [20,21].

## 3. Venom Composition

Like many venomous lineages, the characterisation of sea anemone venom toxins has been done opportunistically, focusing on toxins and taxa that are easily accessible and of potential therapeutic relevance. As a result, the composition of most sea anemone venoms remains unknown despite decades of research [22,23]. Nevertheless, sea anemone venoms have been shown to be complex mixtures of proteins, peptides, and non-proteinaceous compounds. The main components found in sea anemone venom are traditionally grouped into four functional types: (1) phospholipase A_2_ that degrades membrane phospholipids of neuronal and muscle cells, causing nerve damage and muscle inflammation [24]; (2) cytolysins that act on cell membranes and cause cell lysis [25]; (3) neurotoxins that interact with receptors, voltage-gated and ligand-gated ion channels (some of which also have protease inhibitor activity—see Section 3.4.6) [22,26,27,28], thereby altering neural transmission [29,30]; (4) non-proteinaceous compounds (e.g., purines, biogenic amines) that are believed to induce pain during envenomation.

Until recently, no systematic nomenclature existed for naming and organising sea anemone toxins. This resulted in multiple names being assigned to the same toxin, toxins from unrelated species being designated by the same name, and ambiguous name designations. However, in 2012, two articles were published that suggested a rational nomenclature for naming sea anemones toxins. Kozlov and Grishin [31] suggested a nomenclature for cysteine-rich polypeptides toxins from sea anemones, while Oliveira and colleagues [32] suggested a more general nomenclature for naming any kind of sea anemone toxin. Moreover, Norton [33] suggested that in order to avoid confusion with sea anemone toxins and other anemone venom peptides, the nomenclature could be modified to include toxin type, such as Types K1, K2, and K3 for potassium channel toxins.

Here, the criteria stipulated by Oliveira et al. [32] are followed because they can be used for all types of toxins, the species can be identified, and it avoids confusions with similar names. This sea anemone toxin nomenclature is similar to that previously proposed for spider toxins [34] and adapted for centipede toxins [35]. The nomenclature consists of five terms: the first term is a Greek symbol that serves as a broad activity descriptor denoting the molecular target. The second term is a generic name indicating the taxonomic family. The third term is a three-letter code signifying the species of origin, and consists of an initial uppercase corresponding to the first letter of the genus, followed by two lowercase letters that indicate species. The two last terms are formed by an alphanumeric descriptor to assign the chronological order of sequence deposition into public database or original publication related to the toxin. The numerical character indicates the paralogous relationship which is assigned based on amino acid sequence analysis. Within the same species, toxins sharing a high level of sequence identity and similarity are clustered in an isoform or isotoxin group. In each isotoxin group, a lowercase Latin letter is given in alphabetic order according to the date of the toxin sequence published. The nomenclature is not italicized, has hyphens to separate the first three terms, and relies on the taxonomic sea anemone names included in the Hexacorallians of the Word database (http://geoportal.kgs.ku.edu/hexacoral/anemone2/index.cfm) to generate a three-letter code for designating sea anemone species.

Sea anemone toxins have traditionally been classified according to their activity, similarity of amino acid sequence, and the pattern of disulfide bridges (number and distribution of cysteines) [29]. In this way, the toxins are first divided by their molecular target and then by types according to their similarity and mechanism of action (Table 1). However, it is known that proteins and peptides with certain structural characteristics have been more often recruited into venoms and subsequently undergone functional radiation [36]. In most cases, the stabilisation of these molecular scaffolds through disulfide bonds facilitates modifications of non-structural residues, allowing alterations of surface-exposed residues without affecting the structural core [37]. This means that the current classification system suffers from vulnerability due to functional convergence (e.g., toxins with Kunitz or defensins scaffolds both interact with voltage-gated potassium (K_V_) channels) as well as functional promiscuity (e.g., APETx2 interacts with both acid-sensing ion channels (ASICs) and voltage-gated potassium (K_V_) channels). In addition, for most known sea anemone toxins, the exact receptors they target (e.g., ion channel subtypes) is either unknown, or at best incomplete. Because of this, the following discussion on the main components of sea anemone venoms is not divided according to the traditional toxin types. Instead distinctions are made firstly between proteins and non-proteinaceous compounds, secondly between enzymes and other proteins without enzymatic activity, then according to the structural scaffold, and finally according to molecular targets.

### 3.1. Non-Proteinaceous Venom Components

Sea anemones are known to be a rich source of protein and peptide toxins. In contrast, little is known about the non-peptidic components of their venoms. The first small molecule described from a sea anemone was caissarone, a purine derivative isolated in 1986 from the Brazilian sea anemone *Bunodosoma caissarum* [38]. Caissarone induced twitching in electrically stimulated guinea pig ileum-myenteric plexus, and this was attributed to antagonistic actions on the adenosine receptor [39]. Years later, several groups began investigating small-molecule fractions based on a study reporting the antagonism of glutamate receptors by a low molecular weight fraction from venom of the Caribbean sea anemone *Phyllactis flosculifera* [40]. These studies led to isolation of bunodosine 391, an acylated amino acid, from the venom of the Brazilian sea anemone *Bunodosoma cangicum*, which likely acts on 5-hydroxytryptamine (5-HT; serotonin) receptors. Bunodosine 391 is analgesic in animal models of pain, and this activity is blocked by methysergide, a nonselective 5-HT receptor antagonist, but not the opioid receptor antagonist naloxone. Aside from these studies, small molecules from sea anemone venoms remain largely unexplored.

### 3.2. Enzymes

There is a large discrepancy between the types of enzymes reported from transcriptomic studies of sea anemones with those reported from studies on milked venom. As previously mentioned, sea anemones do not have a centralised venom gland, and it is therefore difficult using transcriptomic techniques to distinguish between enzymes with a housekeeping function and those that play a role in envenomation [41]. Because of this uncertainty, only enzymes purified from sea anemone venom will be described in this chapter. So far, PLA_2_, serine protease, and endonuclease D are the only enzymes identified in milked sea anemone venom.

PLA_2_ catalyses the hydrolysis of phospholipids into free fatty acids and lysophosholipids. As phospholipids are one of the main chemical constituents of the cell envelope, enzymes capable of hydrolysing these molecules, such as PLA_2_, can cause membrane disruption. PLA_2_s have been convergently recruited into the venoms of numerous venomous taxa including bees, centipedes, lizards, sea anemones, snakes, spiders, and wasps [42]. The PLA_2_ superfamily currently contains 15 separate groups and numerous subgroups [43], and all of the venom PLA_2_s belong to the group of secreted low molecular weight PLA_2_s. Sea anemone PLA_2_s remain poorly studied. Only few sequences have been reported for PLA_2_s isolated sea anemone venom so far [44]. However, several studies have shown that sea anemone extracts (whole animal, tentacles, acontia) contain PLA_2_ activity. Purification and sequencing of the proteins responsible for these activities will be important to classify and understand the ecological importance of PLA_2_ in sea anemone venom.

### 3.3. Non-Enzymatic Proteins – Cytotoxins

Cytolysins are a group of sea anemone toxins that form pores in cell membranes, and therefore belong to a larger group of ‘pore forming toxins’ (PFTs) [45]. The formation of pores by PFTs can be achieved by inserting either α-helices or β-hairpins within the cell membrane. These proteins exhibit dual behaviour at the water-membrane interface. In water, they remain mostly monomeric and stably folded but, upon interacting with lipid membranes of specific composition, they become oligomeric structures. So far, cytolysins have been isolated from >30 different sea anemones species. Based on their primary structure and functional properties, cytolysins have been classified into five types (types I–V).

Type I cytolysins are 5–8 kDa peptides that form pores in phosphatidylcholine membranes, and additionally have antihistamine activity. So far, this type has been reported in just a few species, such as *Tealia felina* (accepted name *Urticina feline*) [46] and *Heteractis crispa* (previously known as *Radianthus macrodactylus*) [47]. RmI (Δ-stichotoxin-Hcr1e, 5.1 kDa) and RmII (Δ-stichotoxin-Hcr1b, 6.1 kDa) are cytolysins from *H. crispa* that have been biochemically characterized to some extent. They are basic peptides (pI ~ 9.2) that contain cysteine residues but lack tryptophan. However, the structural features of this group of cytolysins are unknown.

**Figure 2:**
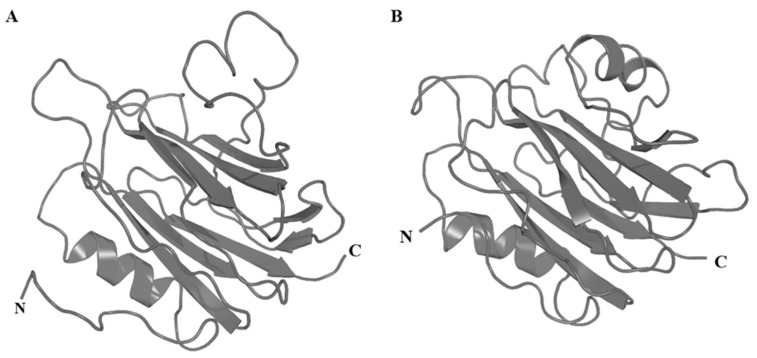
3D structures of actinoporins as exemplified by (**A**) Δ-stichotoxin-She1a (Sticholysin I; PDB accession code 2KS4) and (**B**) Δ-actitoxin-Aeq1a (Equinatoxin II; PDB 1KD6). The N- and C-termini are labelled.

Type II cytolysins are the most abundant and best studied cytolysins from sea anemone venoms. They consist of the family of the α-pore-forming toxins (α-PFTs) [48], which are also known as actinoporins due to their ability to bind membrane phospholipid domains of the host organism and form cation-selective pores [49]. Actinoporins are comprised of a single domain (~20 kDa) that lacks cysteine residues and is equipped with functionally important regions that are conserved throughout the toxin gene family [50,51]. These regions include a prominent patch of aromatic amino acids located on the bottom of the molecule that appear to form the main site of initial binding to membranes [52,53]. The three-dimensional (3D) structure of actinoporins is composed of a tightly folded β-sandwich core flanked on two sides by α-helices (Figure 2), the first of which is encompassed by the first 30 residues and plays a crucial role in pore formation.

Several residues have been manipulated to identify functionally important regions within actinoporins [54], revealing an aromatic-rich region that forms the phosphocholine (POC) binding site, with a single amino acid residue (W112 in Equinatoxin II (EqII or Δ-actitoxin-Aeq1a)) playing a key role in initiating sphingomyelin recognition and pore formation [54,55]. Although the events leading to oligomerisation remain uncertain, both the RGD domain (R144, G145, and D146 in EqII) and a single valine residue (V60 in EqII) are thought to direct protein attachment and play a key role in this process [56]. Finally, a key arginine residue (R31 in EqII) and various hydrophobic residues in the α-helix within the N-terminal region are involved in cell membrane penetration and formation of the ion conduction pathway (discussed below) [48].

Type II cytolysins appear as multigene families [57,58], resulting in many protein isoforms with variable levels of sequence identity (60–80%). Interestingly, these sequence differences are sufficient to cause large differences in solubility as well as hemolytic [59] and general cytolytic [60] activity, which suggests that they may target a wide range of tissues, organisms, or even play several ecological roles. Indeed, several actinoporins are specifically expressed in tissues with discrete functions, including tentacles (suggesting a role in prey capture), mesenterial filaments (suggesting a role in digestion), and column (suggesting a role in defence) [61]. This diversity of activities and functions suggests that the amino acid sequence across actinoporins may have been diversified in order to target cell membranes in specific tissues or animal lineages [25,62].

Given their different membrane selectivity, relatively small size, and lack of cysteines, actinoporins have attracted attention as interesting and convenient models for studying the underlying mechanisms of how PFTs become integral membrane proteins. Although the molecular details of the mechanism by which the actinoporins so potently form pores in target membranes remain elusive, several hypotheses have been proposed. One of these hypotheses suggests that the toxin first attaches to the membrane via specific recognition of sphingomyelin using the aromatic-rich region and adjacent POC binding site [52,53,55,63], where the N-terminal segment of the toxin is then transferred to the lipid–water interface [53,64,65]. Finally, the toxin oligomerises on the surface of the membrane and α-helices from three or four monomers insert into the membrane and form a cation-selective conduction pathway of diameter 1–2 nm [64,66,67,68]. This last step includes an important contribution by membrane lipids and the monomers are likely arranged in a so-called toroidal pore arrangement [69,70]. More comprehensive information is available in a number of specific papers for actinoporins [71,72,73,74,75].

Type III cytolysins are 25–45 kDa PLA_2_, with or without enzymatic activity. They were first detected in the venom of *Aiptasia pallida* [76] and later in venom of sea anemones from the genus *Urticina* (*U. crassicornis* and *U. piscivora*) [77,78]. In contrast with the actinoporins, these cytolysins contain several cysteine residues [77]. They cause hemolysis of mammalian red blood cells at concentrations as low as 10^−10^ M. Interestingly, the hemolytic activity of Type III cytolysins is inhibited by sphingomyelin, but not by cholesterol, which is not common for actinoporins [79].

The Type IV cytolysin family currently consists of a single protein isolated from sea anemone homogenate as a cholesterol-inhibitable cytolysin [80]. This toxin, metridiolysin from *Metridium senile* [25], has a molecular mass of 80 kDa and, similarly to a group of bacterial toxins, is activated by thiols to produce ring structures on membranes [81]. Metridiolysin binds non-specifically to lipid membranes [25] and forms fluctuating K^+^-permeable pores in planar lipid membranes [25]. However, little is known about its biochemical properties or taxonomic distribution outside *M. senile*.

Type V cytolysins are similar to the membrane-attack complex/perforin (MACPF) family of proteins, and they were first discovered in nematocysts of the stinging sea anemone *Phyllodiscus semoni*. MACPF family proteins were originally identified as pore-forming factors, called perforins, utilised in the mammalian host defence immune system [82,83]. Similar to perforins, Type V cytolysins have an EGF-like domain close to the MACPF domain, but they lack the C2 domain for attachment to lipid membranes. Type V cytolysins were the first MACPF proteins found in non-mammalian species, and the first reported case of MACPF proteins recruited into venom. Sea anemone MACPF-cytolysins have a mass of ~60 kDa. So far, only three have been described (AvTx-60A from *Actineria villosa*, and PsTx-60A and PsTx-60B from *Phyllodiscus semoni*), but they are predicted to be present in *Nematostella vectensis* based on its genome sequence [84]. The discovery and characterisation of MACPF-cytolysins in sea anemones has aided our understanding of the mechanism of membrane permeabilisation by MACPF proteins as well as the evolution of MACPF superfamily.

### 3.4. Non-Enzymatic Proteins – Neurotoxins

Neurotoxins are toxins that interfere with the transmission of nerve impulses by modifying the function of ion channels in nerve or muscle cells [85]. Diverse venomous animals have evolved neurotoxins that interact with ion channels to immobilise prey and/or deter predators. Because sea anemones are sessile animals, venom neurotoxins play a critical role in the immobilisation of prey and defence against predators. Neurotoxins are among the best characterised components of sea anemone venoms in terms of their mechanisms of action. They interact with a wide range of ion channels, including ASICs [86,87], TRP channels [22,26,27,28], Na_V_ channels [88,89,90,91] and K_V_ channels [92,93,94]. Of these, K_V_ toxins comprise 136 of the 320 sea anemone toxins reported in UniProtKB [95] and thereby constitute the most diverse group, with Na_V_ toxins in close second place. Cysteine-rich peptides make up virtually all sea anemone neurotoxins, and the diversity of structural scaffolds among these is remarkable. To date, nine unique structural folds have been identified based on 3D structure and/or cysteine-pattern: *Anemonia sulcata* toxin III (ATX-III), β-defensin-like, boundless β-hairpin (BBH), epidermal growth factor-like (EGF-like), inhibitor cystine-knot (ICK), Kunitz-domain, proline-hinged asymmetric β-hairpin (PHAB), small cysteine-rich peptides (SCRiPs), and ShK.

#### 3.4.1. ATX III

**Figure 3:**
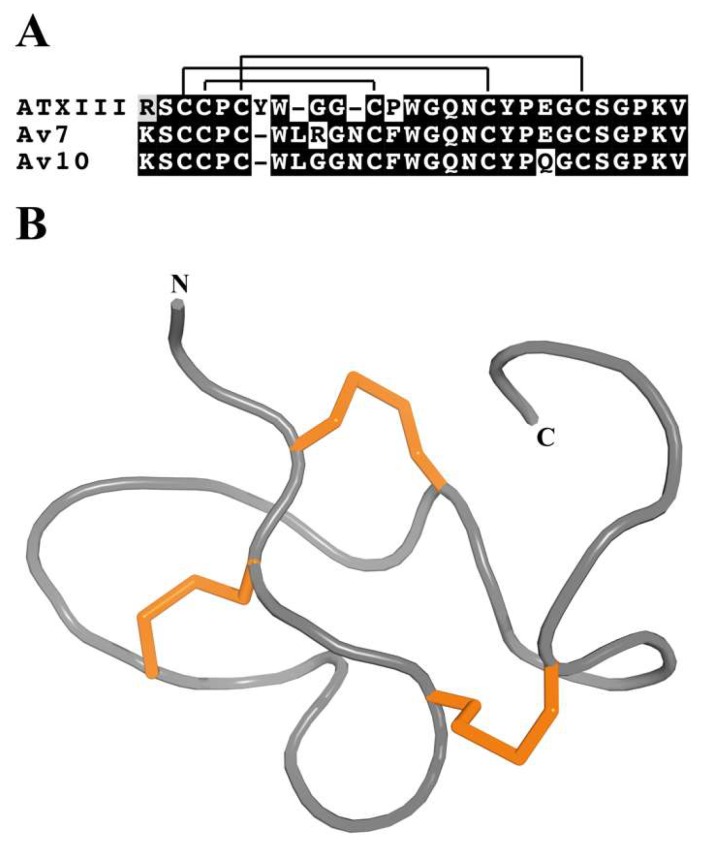
Representative sea anemone toxins with an ATX III motif. (**A**) Alignment of representative sea anemone toxins that adopt the ATX III motif. Disulfide bridge connectivities are indicated above the sequence alignment. Amino acid identities (black boxes) and similarities (grey boxes) are shown. The representative toxin sequences shown are ATX III (δ-AITX-Avd2a; UniProt P01535), Av7 (δ-AITX-Avd2b 3; UniProt C3TS10), and Av10 (δ-AITX-Avd2c; UniProt C3TS07). (**B)** 3D structure of the sea anemone toxin Av3 (PDB accession code 1ANS). The three disulfide bonds are represented by orange tubes.

The ATX III fold is named after the first toxin described with this fold, namely *Anemonia sulcata* toxin III (ATXIII; δ-AITX-Avd2a) [96]. Toxins with this fold form a compact structural motif composed of 27–32 residues with three disulfide bonds; the structure is formed by turn-based secondary-structure elements with a complete absence of α-helices and β-strands (Figure 3) [30,96]. To date, only six ATX III toxins have been identified from three species (*Anemonia viridis*, *Dofleinia armata* and *Entacmaea quadricolor*). All of these toxins delay the inactivation of Na_V_ channels, and several residues located on the surface of the toxins form a hydrophobic patch that may constitute part of the Na_V_ channel binding surface [96]. These toxins are inactive against mice [97,98], but are highly active on insects and crustaceans [99].

#### 3.4.2. β-Defensins

**Figure 4:**
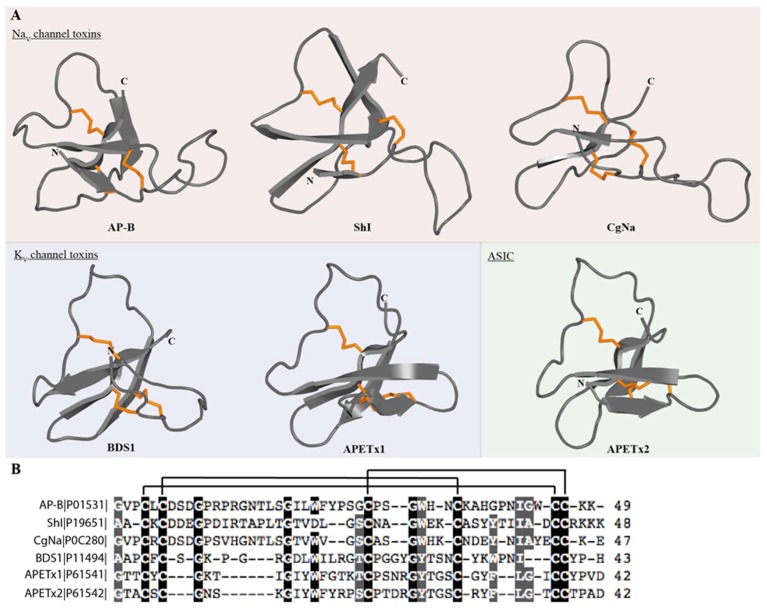
Sequence and structure of sea anemone toxins that adopt a β-defensin fold. (**A**) Representative 3D structures of sea anemone toxins that adopt a β-defensin fold. Disulfide bonds are shown as orange tubes. Toxins are grouped according to their molecular targets. (**B**) Alignment of representative sea anemone toxins that contain a β-defensin motif. Disulfide connectivities are indicated above the sequence alignment. Identical and similar amino acid residues are highlighted in black and grey, respectively. The representative toxin sequences shown are AP-B (Δ-actitoxin-Axm1b, UniProt P01531), ShI (Δ-stichotoxin-She1a, UniProt P19651), CgNa (Δ-actitoxin-Cgg1a, UniProt P0C280), BDS1 (Δκ-actitoxin-Avd4a, UniProt P11494), APETx1 (κ-actitoxin-Ael2a, UniProt P61541), APETx2 (π-actitoxin-Ael2b, UniProt P61542).

The β-defensin-fold generally consists of a short helix or turn followed by a small twisted antiparallel β-sheet (Figure 4A). The six cysteine residues form a characteristic pattern with the first four cysteines being interspaced single cysteines and the last two cysteines forming a pair (CCX_n_, where *n* ≥ 1) near the C-terminus. These cysteines are paired in a C_1_–C_5_, C_2_–C_4_, C_3_–C_6_ fashion to form disulfide bonds that are crucial for maintaining the compact core configuration of β-defensins. β-defensins are antimicrobial peptides that are secreted as part of the innate immune response in a wide range of taxa [100,101]. However, in sea anemone venoms, β-defensin-like peptides have become weaponised to serve as neurotoxins that modify the activity of voltage- and ligand-gated ion channels; this family of peptides includes K_V_ type 3, Na_V_ types 1, 2 and 4, and ASIC toxins [102,103,104,105] (Figure 4).

K_V_ type 3 sea anemone toxins are comprised of 42–43 residue β-defensins, and include among others APETx1 (κ-actitoxin-Ael2a) from *Anthopleura elegantissima* [106] and Blood Depressing Substance I (BDS-I; Δκ-actitoxin-Avd4a) and BDS-II (Δκ-actitoxin-Avd4b) from *Anemonia sulcata* [107]. BDS toxins were first characterized as antihypertensive and antiviral compounds [108]. BDS-I and BDS-II have a 93% sequence identity, and they both inhibit K_V_3.1, K_V_3.2 and K_V_3.4 at nanomolar concentration [107,109]. Both toxins induce a depolarising shift in the voltage-dependence of activation of K_V_3.1 and K_V_3.2, and at high concentrations they weakly inhibit K_V_1.1–5, K_V_2.1–2, K_V_4.1 and K_V_4.3 channels [92]. Despite their classification as K_V_ channel toxins, it was recently shown that some K_V_ type 3 toxins, including BDS-I, also interact with Na_V_ channels [110,111]. BDS-I is capable of modulating Na_V_ channel gating in a manner similar to previously known neurotoxin receptor site 3 sea anemone toxins, such as Na_V_ type 1 and 2, but with different subtype selectivity [110].

Although they are both classified as K_V_ type 3 toxins and they assume the same 3D fold, APETx1 only has ~40% sequence identity with BDS-I/-II and they have different activity on K_V_ channels. APETx1 is a potent blocker (IC_50_ 34 nM) of the human ether-a-go-go-related K^+^ channel (hERG). The toxin induces a shift in the voltage dependence of both activation and inactivation, resulting in a block of potassium currents [106]. APETx1 also inhibits Na_V_1.2–Na_V_1.6 and Na_V_1.8 [111]. Contrary to its modulation of hERG, APETx1 does not alter the voltage dependence of activation or steady-state inactivation of Na_V_ channels.

**Figure 5:**
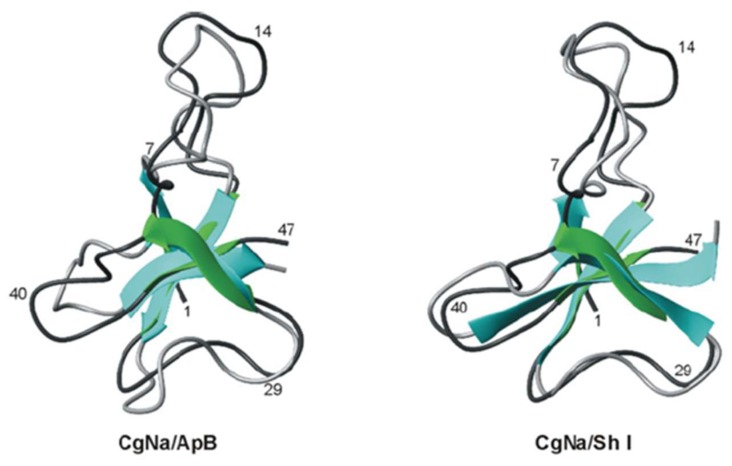
Superimposed cartoon representation of the structures of CgNa (Δ-actitoxin-Cgg1a) and ApB (left) and CgNa and ShI (Δ-stichotoxin-She1a) (right). CgNa is coloured green and dark grey. Figure modified with permission from Biochemical Journal; published by Portland Press, 2007 [118].

Na_V_ type 1 and 2 are the largest Na_V_ channel toxins from sea anemones, with 46–54 residues. Although they are divided into two types, they have up to 50% sequence identity. Moreover, because certain toxins from *Halcurias sp., Nematostella vectensis* and *Condylactis gigantean* resemble both type 1 and 2 sequences [112,113], Moran and co-workers [88] have suggested that this classification should be revaluated (Figure 5). The 3D structures of both type 1 and 2 consist of an antiparallel β-sheet composed of four β-strands and a highly flexible loop, which has been named the ‘Arg14 loop’, because Arg14 is the most conserved residue (Figure 5) [114,115,116,117,118]. Site-directed mutagenesis of AP-B (Δ-actitoxin-Axm1b) revealed that the flexibility of this loop is important for the selectivity and binding of these toxins to Na_V_ channels.

Na_V_ type 1 sea anemone toxins are highly potent modulators of Na_V_ channels. These toxins bind to a region of the channel named receptor site-3 (i.e., the extracellular region of the voltage sensor in channel domain IV), which is also recognised by scorpion α-toxins [119]. Given the close evolutionary relationship between crustaceans (sea anemone prey and predators) and insects (which constitute prey to several intertidal sea anemones [120]), sea anemone toxins also have a profound effect on insect Na_V_ channels. Na_V_ type 1 toxins have therefore been considered as lead compounds in the development of bioinsecticides [90]. Anthopleurins (type 1 Na_V_ channel toxins isolated from the genus *Anthopleura*) and related type 1 Na_V_ channel toxins have also been considered for cardiovascular therapeutic applications, but this development has been hindered by arrhythmogenic activity in the heart [121].

The Na_V_ type 4 sea anemone toxin family is comprised of only two isotoxins, calitoxin I (Δ-hormotoxin-Cpt1a) and calitoxin II (Δ-hormotoxin-Cpt1b) from *Calliactis parasitica* [122,123]. These toxins contain 46 residues with only a single amino acid difference (Glu8 versus Lys8). In crustacean nerve muscle preparations, they interact with axonal, but not muscle, membranes, inducing a massive release of neurotransmitter that causes strong muscle contractions. They resemble Na_V_ type 1 and 2 toxins with regard to chain length and the number of disulfide bridges (three) but not in amino acid sequence, with only ~45% sequence identity. Evaluation of the Na_V_ subtype selectivity of these toxins and isolation of more members of this type should help in future classification of this group.

APETx2 (π-actitoxin-Ael2b) from *Anthopleura elegantissima* was the first ASIC-targeting peptide isolated from sea anemone venom and only the second from any venomous animal [105]. APETx2 is a 4558 Da peptide (42 residues) that selectively blocks ASIC3 homomeric channels (IC_50_ 63 nM) and the ASIC3-containing heteromers ASIC2b-ASIC3 (IC_50_ 117 nM), ASIC1b-ASIC3 (IC_50_ 0.9 µM) and ASIC1a-ASIC3 (IC_50_ 2 µM). The structure of APETx2 was determined using two-dimensional ^1^H NMR spectroscopy using the native toxin [103] (Figure 4). It belongs to the disulfide-rich all-β structural family with a fold typical of the β-defensin family [124]. To date, three peptides have been isolated from sea anemone venoms that target ASIC channels and, interestingly, they do not have the same 3D fold [125]. This structural diversity highlights sea anemone venoms as excellent sources of novel ion channel modulators.

#### 3.4.3. Boundless β-Hairpin

**Figure 6:**
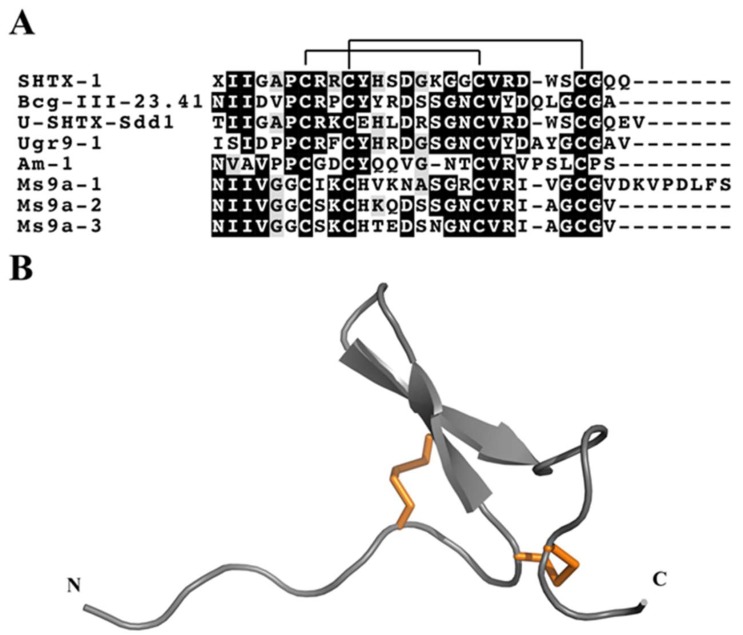
Representative sea anemone toxins with BBH mortif. (**A**) Alignment of representative sea anemone toxins that adopt the BBH motif. Disulfide connectivities are indicated above the sequence alignment. Amino acid identities (black boxes) and similarities (grey boxes) are highlighted. The representative toxin sequences shown are SHTX-1 (κ-stichotoxin-Shd1a; UniProt P0C7W7), Bcg-III-23.41 (U-BcgTx1a; UniProt P86467), U-SHTX-Sdd1 (UniProt C0HJB4), Ugr9-1 (π-AnmTX Ugr 9a-1; UniProt R4ZCU1), Am-1 (Δ-AITX-Amc1a; UniProt P69929), Ms9a-1 (T-AnmTX Ms 9a-1; UniProt C0HK13), Ms9a-2 (T-AnmTX Ms 9a-2; UniProt C0HK13) and Ms9a-3 (T-AnmTX Ms 9a-3; UniProt C0HK13). (**B**) 3D structure of the sea anemone toxin Ugr9-1 (PDB 2LZO). The two disulfide bonds are represented by orange tubes.

Osmakov and collegues [126] isolated three peptides with uncommon β-hairpin-like structure from the venom of the sea anemone *Urticina grebelnyi*. One of these peptides, Ugr9-1 (π-actitoxin-Ugr1a), reversibly inhibits both transient and sustained currents mediated by human ASIC3 channels. NMR spectroscopy revealed that Ugr9-1 has an unusual structure, stabilised by two disulfide bonds, with three classical β-turns and a twisted β-hairpin devoid of inter-strand disulfide bonds (Figure 6B). Although the authors suggested that this represents a novel peptide fold, which they named the boundless β-hairpin (BBH), other sea anemone toxins with similar disulfide framework had in fact been reported previously [127,128]. These toxins belong to K_V_ type 4, which is comprised of three toxins, Bcg-III-23.41 (U-BcgTx1a) and SHTX-1/SHTX-2 (κ-stichotoxin-Shd1a/b) (Figure 6A). The activity of SHTX-I was indirectly assayed by competitive inhibition of the binding of ^125^I-α-dendrotoxin to rat synaptosomal membranes but its channel blocking specificity is not yet known. The only difference between SHTX-I and II is a posttranslational modification of Pro6 in SHTX-II to a hydroxyproline in SHTX-I.

Another toxin with a similar framework was discovered in the venom of *Stichodactyla duerdeni* and named U-SHTX-Sdd1. Although its pharmacological activity remains to be determined, U-SHTX-Sdd1 was the first sea anemone toxin described with an *O*-linked hexose-*N*-acetyl posttranslational modification, in this case of the N-terminal threonine [129]. Recently, a novel BBH peptide that produces a significant potentiating effect on allyl isothiocyanate- and diclofenac-induced TRPA1 currents was isolated from venom of the sea anemone *Metridium senile* [130]. Ms 9a-1 (τ-metridiotoxin-Ms9a) acts as a positive modulator of TRPA1 in vitro, but did not cause pain or thermal hyperalgesia when injected into the hind paw of mice. Interestingly, the mature Ms 9a-1 is post-translationally liberated from two near identical prepropeptides, encoded by ms9.1 and ms9.2, that each also include the additional mature peptide domains Ms 9a-2 (U-metridiotoxin-Ms9b) and Ms 9a-3 (U-metridiotoxin-Ms9c), respectively. These second domain peptides are distinguished from Ms 9a-1 by a shorter C-terminal tail and a non-homologous region between the 2nd and 3rd Cys residues (Figure 6A).

#### 3.4.4. EGF-Like Peptides

Gigantoxin I (ω-stichotoxin-Sgt1a) is a peptide toxin from *Stichodactyla gigantea* that has homology with mammalian epidermal growth factor (EGF). In accordance with this sequence homology, this toxin exhibits EGF activity as evidenced by the rounding of human epidermoid carcinoma A431 cells [131]. Gigantoxin I also modulates the activity of TRPV1 channels [28], but this activity results from the involvement of the EGF receptor/PLA_2_/arachidonic acid/lipoxygenase pathway in indirect activation of TRPV1. Gigantoxin I was the first toxin described that induced this effect, and this group of toxins might thus help generate a better understanding of the regulation of TRPV1 channels [28].

#### 3.4.5. Inhibitor Cystine Knot Fold

**Figure 7:**
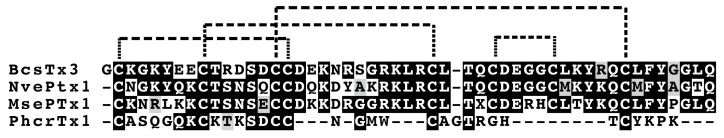
Alignment of sea anemone toxins that likely adopt an ICK fold. Predicted disulfide connectivities are indicated. Amino acid identities (black boxes) and similarities (grey boxes) are highlighted. The representative toxin sequences shown are BcsTx3 (κ-actitoxin-Bcs4a; UniProt C0HJC4), NvePTx1 (U-EWTX-NvePTx1; UniProt A7RMN1), MsePTx1 (U-metritoxin-Msn2a; UniProt P0DMD7) and PhcrTx1 (π-phymatoxin-Pcf1a; UniProt C0HJB1).

The inhibitor cystine knot (ICK) motif is one of the most widely recruited peptide folds in venomous animals [132]. Although the 3D structure of putative sea anemone ICK toxins remain to be confirmed, two different types of sea anemone toxins display cysteine patterns characteristic of the ICK fold, namely Kv type 5 toxins and the ASIC toxin PhcrTx1 (π-phymatoxin-Pcf1a) [87]. PhcrTx1 was the first peptide characterized from the venom of *Phymanthus crucifer*. It is a 32-residue peptide with three disulfide bonds (Figure 7) that reversibly inhibits ASIC currents in rat dorsal root ganglia neurons with an IC_50_ of 100 nM [87]. Although its disulfide framework and 3D structure remain to be determined, the distribution of cysteines has the “classic” ICK signature (i.e., CX_n_CX_n_CCX_n_CX_n_C, where X is any amino acid and *n* indicates a variable number of amino acid residues). This cysteine pattern is also characteristic of the only K_V_ type 5 toxin reported to date, namely BcsTx3 (κ-actitoxin-Bcs4a) from *B. caissarum* [93]. BcsTx3 is a single-chain 50-residue peptide with high affinity for *Drosophila* Shaker IR channels (IC_50_ 94 nM) over K_V_1.2 (IC_50_ 173 nM), K_V_1.3 (IC_50_ 1007 nM), and K_V_1.6 (IC_50_ 2246 nM) channels. It is internally crosslinked by four disulfide bridges, three of which likely form a classic ICK framework, but it differs from PhcrTx1 by the presence of an additional disulfide bond that likely stabilises loop three. In addition to PhcrTx1 and BcsTx3, there are putative ICK peptides in the venom of *Nematostella vectensis* (NvePTx1) and *Metridium senile* (MsePTx1). Based on cysteine pattern and sequence similarity, they are potential new members of the K_V_ type 5 toxins family, but their presence in venom remains to be confirmed (Figure 7).

#### 3.4.6. Kunitz-Domain

**Figure 8:**
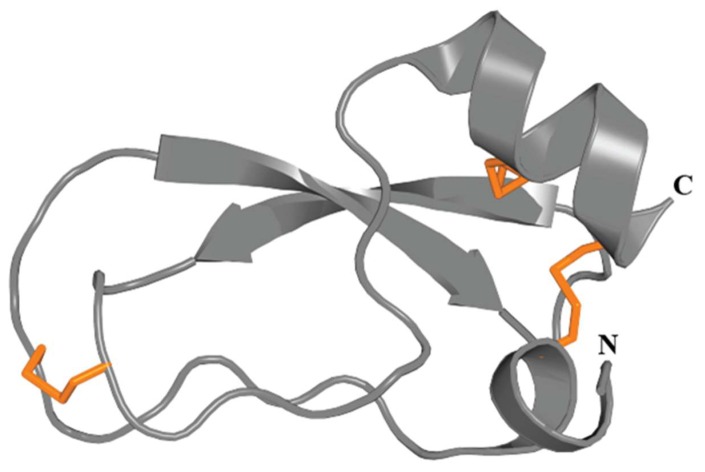
3D structure of the K_V_ type 2 sea anemone toxin ShPI-I (π-stichotoxin-She2a) (PDB 3OFW). Peptides of this type are homologous to Kunitz-type serine protease inhibitors. The three disulfide bonds are represented by orange tubes.

The Kunitz-type protease inhibitors are the best-characterized family of serine protease inhibitors, probably due to their abundance in several organisms. The Kunitz-type motif consists of ~60 amino acid residues stabilised by three disulfide bridges with C_1_–C_6_, C_2_–C_4_, C_3_–C_5_ connectivity. The 3D structure is characterized by an α/β/α motif [133] (Figure 8) with a hydrophobic core. The first reports on the existence of protease inhibitors in sea anemones date from the 1970s [134,135]. To date, protease inhibitor peptides and neurotoxins have been isolated from sea anemone’s whole bodies, tentacles, secreted mucus, and aggressive organs such as acrorhagi, which are present in some species from the family Actiniidae [136]. Several protease inhibitors have already been isolated or partially purified and characterised from several sea anemone species.

K_V_ type 2 sea anemone peptide toxins block K_V_1 channel currents, although with much less potency than K_V_ type 1 toxins [30,137,138]. Their biological role is unclear. It is supposed that these protease inhibitors could: (1) protect sea anemones from prey/predator proteases; (2) protect the toxins injected into prey or predators from degradation by host proteases; (3) act on the regulation of digestive mechanisms, including self-digestion by their own enzymes or those of symbiotic microorganism; (4) due to their dual inhibition of protease and K_V_ channels, they could also be used to paralyse prey [139]. The sea anemone kalicludines (AsKC1 to AsKC3, κπ-actitoxin-Avd3b-d) from *Anemonia sulcata*, APEKTx1 (κπ-actitoxin-Ael3a) from *Anthopleura elegantissima*, SHTX-3 (κπ-stichotoxin-Shd2a) from *Stichodactyla haddoni*, and Sh1 (δ-HTX-She1a) from *Stichodactlyla helianthus*, are examples of toxins that inhibit both proteases and K_V_ channels [128,138,140].

Many protease inhibitors were isolated from a body extract of *Heteractis crispa* (previously *Radianthus macrodactylus*), but only a few have been fully characterised. Protease inhibitors were obtained from a water-ethanol extract of *H. crispa*, and the primary structure was elucidated for one of them, named Kunitz-type trypsin inhibitor IV or Jn-IV (π-stichotoxin-Hcr2a) [141]. Four trypsin inhibitors were subsequently isolated (InI–InIV), one of which (InI) was partially characterised. Later on, a Kunitz-type toxin designated InhVJ (π-stichotoxin-Hcr2e) was also isolated from a *H. crispa* extract [142,143]. InhVJ is highly specific toward trypsin and α-chymotrypsin and does not inhibit other serine (thrombin, kallikrein and plasmin), cysteine (papain), or aspartic (pepsin) proteases.

**Figure 9:**
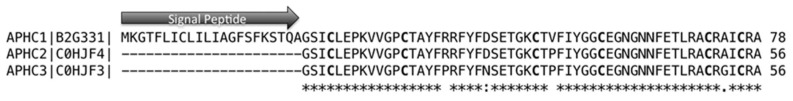
Aligment of APHC1, 2 and 3 (UniProt B2G331, C0HJF3 and C0HJF4, respectively). Cysteines are highlighted in bold, sequence differences are marked with dots, and conserved positions are marked with *.

Recently, APHC1 (τ-stichotoxin-Hcr2b) [27] and two homologous peptides (APHC2 and APHC3, τ-stichotoxin-Hcr2b and -Hcr2c) [144] were characterized from *H. crispa*. The APHC toxins contain 56 residues and have high sequence similarity with the Kunitz-type protease inhibitor family [31]. Consistent with this, APHC1 and APHC3 are weak inhibitors of serine proteases [27,145], although their main pharmacological activity is on TRPV1 channels [27]. TRPV1 was previously described as a pharmacological target of other cnidarians venoms, such as jellyfish [146]. However, APHC1 was the first peptidic TRPV1 modulator isolated from sea anemone venom [27]. The primary structure of APHC1 (UniProt B2G331) and APHC3 (UniProt C0HJF4), differ in only four amino acid residues (Figure 9). These substitutions result in differences in their ability to modulate TRPV1. 200 nM APHC1 inhibits ~32% of capsaisin-induced currents [27] while APHC3 has a lower inhibitory effect (25%) at higher concentrations (300 nM) [147]. Nevertheless, both toxins have antinociceptive and analgesic activity in vivo at doses of 0.01–0.1 mg/kg due to their inhibition of TRPV1. Andreev et al. [147] suggested that APHC1 and APHC3 might represent a new class of TRPV1 modulators that produce a significant analgesic effect without hyperthermia.

#### 3.4.7. Proline-Hinged Asymmetric β-Hairpin (PHAB) Fold

**Figure 10:**
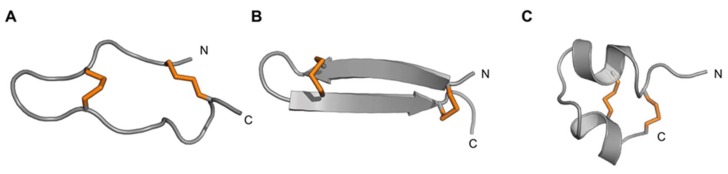
Comparison of the PHAB fold with other peptide folds containing two disulfide bonds and a similar number of residues (16–29 residues). Disulfide bonds are shown as orange tubes and N- and C-termini are labelled. (**A**) Ate1a (PDB 6AZA); (**B**) β-hairpin fold represented by the spider peptide gomesin (PDB 1KFP); (**C**) CS α/α motif represented by the scorpion toxin κ-hefutoxin1 (PDB 1HP9). Figure modified with permission from Cellular and Molecular Life Sciences; published by Springer International Publishing, 2018 [148].

Two unusually short toxins comprised of only 17 amino acid residues were recently described from the venoms of *Actinia tenebrosa* and *A. bermudensis*, and named Ate1a (κ-Actitoxin-Ate1a) and AbeTx1 (κ-Actitoxin-Abe1a), respectively [148,149]. Structural characterisation of the first of these, Ate1a, revealed a previously undescribed fold comprised of a β-hairpin-like topology stabilised by two disulfide bonds [148]. However, the two sides of the hairpin are of unequal length, with the longer side containing a short three-residue proline-hinge that likely prevents the formation of any secondary structure. The consequently named proline-hinged asymmetric β-hairpin (PHAB) fold is thus neither a true hairpin scaffold, nor similar to any other previously described hairpin-like peptide folds such as the β-hairpin antimicrobial peptides (AMPs) [150] (Figure 10) or the cystine-stabilised α/α (CSαα) fold [151] (Figure 10). Both Ate1a and AbeTx1 inhibit specific subtypes of K_V_ channels (K_V_1.1, 1.2 and 1.6), and they thus comprise a 6^th^ type of sea anemone K_V_ toxin.

Although PHAB toxins have only been confirmed to be present in the venoms of *A. bermudensis* and *A. tenebrosa,* they have also been identified in transcriptomes of *Stichodactyla haddoni* and *Anemonia viridis*, suggesting they are widespread in Actinioidea. All PHAB toxins found to date are encoded as multiple repeats of identical mature peptide regions contained on single transcripts. Interestingly, the sequence identities of these mature peptide domains appear to be conserved through intra-gene concerted evolution, which is a highly unusual process that has not been reported for any other toxin gene [148]. A combination of toxicity assays and mass spectrometry imaging (MSI) revealed that Ate1a is almost exclusively localised in tentacles, lacks antimicrobial activity, and probably serves a predatory function via potent inhibition of prey K_V_ channels. The intra-gene concerted evolution of these toxins is therefore likely a mechanism for maintaining effective expression levels of an ecologically important toxin.

#### 3.4.8. SCRiPs

**Figure 11:**
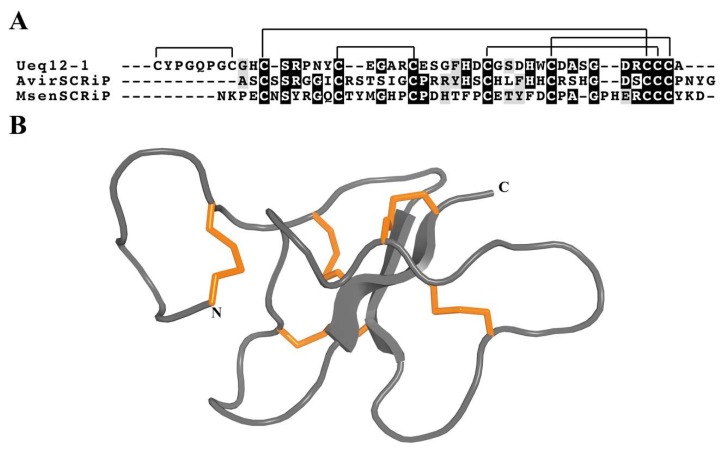
Representative sea anemone toxins with SCRiP motif. (**A**) Alignment of representative sea anemone SCRiP toxins. Disulfide connectivities are indicated above the sequence alignment. Amino acid identities (black boxes) and similarities (grey boxes) are highlighted. The representative toxin sequences shown are Ueq12-1 (τ-AnmTx Ueq 12-1; UniProt C0HK26), AvirSCRiP (UniProt P0DL61) and MsenSCRiP (UniProt P0DL60). (**B**) 3D structure of the sea anemone toxin Ueq 12-1 (PDB 5LAH). The three disulfide bonds are shown as orange tubes.

SCRiPs were originally identified as genes unique to reef-building corals (Scleractinia) that are downregulated during heat stress [152]. Given the similarity of their temporal expression pattern with that of galaxin, a key protein involved in the biomineralisation process [153], SCRiPs were initially implicated in calcification of the coral skeleton [152]. However, Jouiaei et al. [89] showed that SCRiPs from coral reef (*Acropora millepora*) cause profound neurotoxic effects in fish and it is most likely that they are employed as neurotoxins. Moreover, BLAST searches uncovered SCRiP homologues in the sea anemones *Anemonia viridis* and *Metridium senile* [89] (Figure 11). Recently, the first SCRIP was isolated from a sea anemone by Logashina and co-workers [22], who isolated and characterized a peptide from *Urticina eques,* Ueq 12-1 (τ-AnmTx Ueq 12-1). This study confirmed that SCRiPs act as toxins as predicted by Jouiaei and colleagues; Ueq 12-1 was found to be a bifunctional molecule that exhibits both antimicrobial and TRPA1 potentiating activity, producing an analgesic effect in animal models of pain [22].

SCRiPs contain 8–10 cysteine residues, including a characteristic triplet of cysteines near the C-terminus (Figure 11A). The 3D structure of Ueq 12-1 reveals that SCRiPs are organized into a W-shaped structure (Figure 11B), the core of which is formed by a three-stranded antiparallel β-sheet, a small two-stranded parallel β-sheet, and one turn of a 3_10_ helix stabilized by 4–5 disulfide bridges (C1–C2, C3–C8, C4–C7, C5–C9 and C6–C10). The surface of the peptide is polar without pronounced clusters of positively or negatively charged side chains [22].

#### 3.4.9. ShK Motif

**Figure 12:**
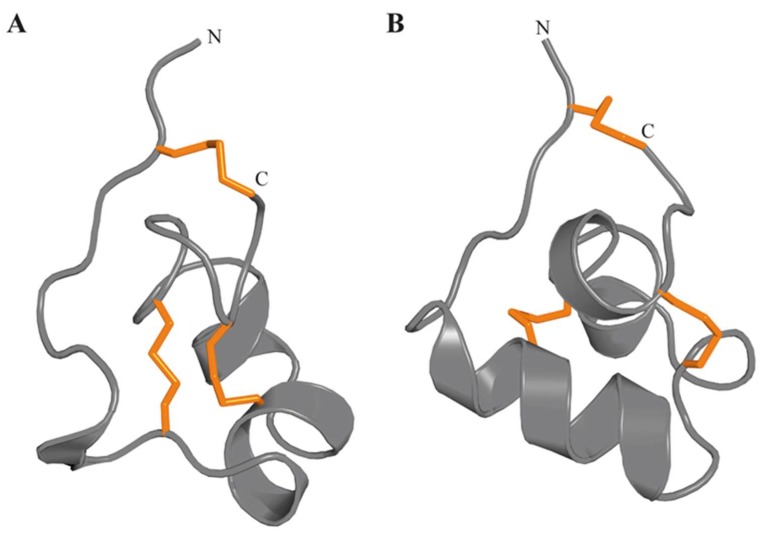
3D structure of K_V_ type 1 sea anemone toxins. (**A**) Structures of BgK (κ-actitoxin-Bgr1a, PDB 1BGK), and (**B**) ShK (PDB 1ROO). The three disulfide bonds are represented by orange tubes.

The ShK motif was named after the toxin ShK (κ-stichotoxin-She3a), which was identified from the venom of *Stichodactyla helianthus*. This toxin belongs to the K_V_ type 1 family, which is comprised of toxins with 35–37 amino acid residues that block K_V_1 channels. The solution structure of ShK [154] consists of two short α-helices encompassing residues 14–19 and 21–24, and an N-terminus with an extended conformation up to residue 8, followed by a pair of interlocking turns that resemble a 3_10_-helix (Figure 12). ShK blocks K_V_1.1, K_V_1.3 and K_V_1.6 channels with picomolar potency, and three other channels (K_V_1.2, K_V_3.2, and Intermediate conductance calcium-activated potassium channel protein 4 (K_Ca_3.1)) with nanomolar potency [155].

The surface of ShK involved in binding to K_V_ channels has been probed using alanine scanning mutagenesis and selected toxin analogues [156]. These studies revealed that two residues, Lys22 and Tyr23, are crucial for ShK activity, as also found subsequently for BgK (κ-actitoxin-Bgr1a) [157], while other residues contribute to the K_V_ channel-binding surfaces. However, Gasparini and colleagues [158] proposed that the Lys-Tyr motif be more broadly defined as a lysine and a neighbouring hydrophobic residue. ShK blocks K_V_ channels by binding to a shallow vestibule at the outer entrance to the ion conduction pathway and occluding ion entrance to the pore [159,160,161]. Although it inhibits several K_V_ subtypes, ShK most potently blocks K_V_1.3 with an IC_50_ of ~10 pM. K_V_1.3 plays a critical role in subsets of T and B lymphocytes implicated in autoimmune disorders, and ShK has therefore been studied as potential immunomodulator for therapy of autoimmune diseases. ShK analogs were developed to be more specific to K_V_1.3 [159]. One of these analogues, ShK-186, is being developed as a therapeutic for autoimmune diseases. ShK-186 (dalazatide) was well tolerated in a recently completed human phase 1A safety trial [162], and it is being advanced by Kineta Inc. (Seattle, WA, USA) into Phase 2 clinical trials [163].

Similar to ShK, BgK inhibits K_V_1.1, K_V_1.2, K_V_1.3, K_V_1.6 and K_Ca_ currents at nanomolar concentrations [157], although it has a highest affinity for K_V_1.1 (*K*_d_ = 6 nM for Kv1.1, 15 nM for Kv1.2, 10 nM for Kv1.3). Beraud and co-workers [164] proposed that K_V_1.1 blockade has broad therapeutic potential in neuroinflammatory diseases (multiple sclerosis, stroke, and trauma). They therefore used an analog of BgK, BgK-F6A, which has the same high affinity for K_V_1.1 (IC_50_ 0.72 nM) but decreased affinities for K_V_1.2 (IC_50_ 400 nM) and K_V_1.3 (IC_50_ 800 nM), to provide preclinical evidence that K_V_1.1 blockers could be used to treat neuroinflammatory diseases.

### 3.5. Others

In addition to the families of sea anemone toxins described above, both known and unknown cysteine frameworks have been identified in proteomic and transcriptomic studies of sea anemone venoms. However, the structure and function of these toxins remain to be determined. For instance, we recently reported endonuclease D, serine protease S1, CAP, WSC domain proteins, and 12 new families of disulfide-rich peptides in the venom of *S. haddoni* [41]. This highlights that there is still much to be learnt about the composition of sea anemone venoms and the role of individual venom components,

## 4. Conclusions

Sea anemone venoms have long been recognised as a rich source of peptides with diverse structure and pharmacological properties. The majority of studies on sea anemone toxins have focused on toxins of medical significance and those with therapeutic potential, and consequently fundamental studies of venom composition and the evolution and ecological role of venom toxins are scarce. It is likely that the growing interest in these venoms combined with the increased taxonomic diversity of these ancient venomous invertebrates being studied will lead to the discovery of unique peptide scaffolds as well as novel and unexpected pharmacological activities.

## Figures and Tables

**Table 1:** Major structural and pharmacological toxin categories in sea anemone venoms. Type I and IV cytolysins have not been included as their structures remain unknown.

Protein Type	Structural Family	Pharmacological Group ^1^
Enzymes	Endonuclease D	Unknown
	Phospholipase type A_2_ (PLA_2_)	PLA_2_ Type III cytolysins
	Serine protease S1	Unknown
*Non*-enzymatic proteins	Actinoporins	Type II cytolysins
	CAP	Unknown
	WSC domain proteins	Unknown
Peptide neurotoxins	ATX-III	Na_V_ type 3
	B-defensin-like	ASIC K_V_ type 3 Na_V_ type 1 Na_V_ type 2 Na_V_ type 4
	Boundless β-hairpin (BBH)	ASIC K_V_ type 4
	Epidermal growth factor-like (EGF-like)	EGF activity TRPV1
	Inhibitor cystine-knot (ICK)	ASIC K_V_ type 5
	Kunitz-domain	K_V_ type 2 TRPV1 Protease inhibitor
	Proline-hinged asymmetric β-hairpin (PHAB)	K_V_ type 6
	Small cysteine-rich peptides (SCRiPs)	TRPA1
	ShK	K_V_ type 1

^1^CAP = CRiSP (cysteine-rich proteins), allergen (Ag-5), and pathogenesis related (PR-1); Na_V_ = voltage-gated sodium channel; TRPA1 = transient receptor potential channel type A1; TRPV1 = transient receptor potential channel type V1.
